# DFT insights into the electronic structure, mechanical behaviour, lattice dynamics and defect processes in the first Sc-based MAX phase Sc_2_SnC

**DOI:** 10.1038/s41598-022-18336-z

**Published:** 2022-08-18

**Authors:** M. A. Hadi, S.-R. G. Christopoulos, A. Chroneos, S. H. Naqib, A. K. M. A. Islam

**Affiliations:** 1 Department of Physics, University of Rajshahi, Rajshahi, 6205 Bangladesh; 2 Faculty of Engineering, Environment and Computing, Coventry University, Priory Street, Coventry, CV1 5FB UK; 3 Department of Electrical and Computer Engineering, University of Thessaly, 38221 Volos, Greece; 4 Department of Materials, Imperial College, London, SW7 2AZ UK; 5 International Islamic University Chittagong, Kumira, Chittagong, 4318 Bangladesh

**Keywords:** Materials science, Physics

## Abstract

Here we employed the density functional theory calculations to investigate some physical properties of first Sc-based MAX phase Sc_2_SnC including defect processes to compare with those of existing M_2_SnC phases. The calculated structural properties are in good agreement with the experimental values. The new phase Sc_2_SnC is structurally, mechanically and dynamically stable. Sc_2_SnC is metallic with a mixture of covalent and ionic character. The covalency of Sc_2_SnC including M_2_SnC is mostly controlled by the effective valence. Sc_2_SnC in M_2_SnC family ranks second in the scale of deformability and softness. The elastic anisotropy level in Sc_2_SnC is moderate compared to the other M_2_SnC phases. The hardness and melting point of Sc_2_SnC, including M_2_SnC, follows the trend of bulk modulus. Like other members of the M_2_SnC family, Sc_2_SnC has the potential to be etched into 2D MXenes and has the potential to be a thermal barrier coating material.

## Introduction

Compounds of the ternary laminated family, discovered six decades ago as the H-phases, are now referred to as the MAX phases^1,2^. This family is chemically represented by M_*n*+1_AX_*n*_, where M is a transition metal, A is an A-group element and X is either carbon or nitrogen or boron^3^. The integer *n* is called the layer index of the M atom. According to *n*, the MAX phase family is divided into six sub-families so far, such as 211, 312, 413, 514, 615, and 716 MAX phases^3^. This family has also been described as metallic ceramics because they possess many metallic and ceramic properties^3^. Similar to metals, some MAX phases are electrically and thermally conductive, resistant to thermal shock, damage tolerant, and readily machinable. Again, they resemble ceramics, as some of them are lightweight, wear resistant, elastically rigid, brittle, and resistant to oxidation and corrosion.

The crystal structure of MAX phases consists of nearly close-packed layers of MX_6_ octahedra interpolated with square-planar slabs of A-atomic layers. In these the X atoms occupy the octahedral sites between the M-atoms. The A atoms reside in the center of trigonal prisms, which are slightly larger than the octahedral sites and can therefore better accommodate the relatively large A-atoms^4^. The interposing pure A-element planes are mirror planes to the zigzagging M_*n*+1_X_*n*_-slabs. Alternatively, the structure of the MAX phases consists of highly symmetrical unit cells that are atomically layered along the c-axis. In the unit cell, the (*n* + 1) ceramic MX-layers are stacked along the c-axis between the two metallic A-layers. The thickness of these atomic layers is within the nanometer range and this is the reason MAX phases are sometimes termed as nanolaminates. The periodic arrangement of the metallic and ceramic layers is the reason for the metallic and ceramic properties of the MAX phases. MAX phases have numerous potential applications ranging from aerospace to nuclear reactors^5^. Recently, MAX phases are used to synthesize the two-dimensional MXenes, which are being used as energy storage materials and as electrodes in electrochemical capacitors, micro-supercapacitors, and batteries^6–9^.

Interest in the Sn-containing MAX phase is considerable in the community because of the report on attractive electrochemical performance of Nb_2_SnC in Li-ion electrolytes^7^. Importantly, two of the three MAX phases discovered after this report are Sn-based MAX Phases. These new members in MAX family are V_2_SnC^10^, Zr_2_SeC^11^, and Sc_2_SnC^12^. The last one is the first Sc-based MAX phase reported with full crystallographic information. Previously, Sc_2_InC was included into a list for H-phases in a paper^13^, however, without any crystallographic data and the source were mentioned as private communication. Up to now there is no experimental evidence for the synthesis and characterization of Sc_2_InC. Therefore, it can be inferred that Sc_2_SnC is the first Sc-based compound in MAX family. On the other hand, there are six 211 MAX carbides containing Sn as A-site element with different M atoms. These are V_2_SnC, Lu_2_SnC, Ti_2_SnC, Nb_2_SnC, Hf_2_SnC and Zr_2_SnC. These phases are studied extensively and their possible applications are predicted in different studies. Kanoun et al. have studied the mechanical, electronic, chemical bonding and optical properties of Ti_2_SnC, Zr_2_SnC, Hf_2_SnC and Nb_2_SnC using DFT^14^. Bouhemadou has conducted a theoretical study of the pressure effect on the structural and elastic properties of M_2_SnC (M = Ti, Zr, Nb, Hf) phases^15^. Hadi et al. have investigated the electronic structures, bonding natures and defect processes in the five Sn-based 211 MAX phases^4^. The mechanical behavior, lattice thermal conductivity and vibrational properties of Lu_2_SnC MAX phase have also been investigated^16^. The V_2_SnC MAX phase is theoretically predicted as a chemically stable, damage and radiation tolerant TBC material^17^. Sc_2_SnC is exceptional among the M_2_SnC phases as its M-element Sc is a rare-earth element, which, in general, in the MAX compounds is typically a transition metal. Therefore, Sc_2_SnC is unique among the M_2_SnC MAX phases. This motivated the present DFT investigation which aims to consider all existing Sn-based 211 MAX phase carbides, so as to understand the role of M-elements on the physical properties of a particular A-atom based MAX carbides. Here we systematically calculated the structural, electronic, mechanical, and thermal properties including Vickers hardness and defect processes of Sc_2_SnC. The derived properties are compared with those found for previously synthesized M_2_SnC MAX phases so as to facilitate comparison and explore the deviation of properties of Sc_2_SnC among the existing M_2_SnC MAX phases.

## Computational methods

The DFT calculations were executed with the CASTEP code^18^. The Perdew-Burke-Ernzerhof (PBE) functional in the frame of generalized gradient approximation (GGA) was employed to estimate the electronic exchange–correlation energy^19^. Ultra-soft pseudo-potential developed by Vanderbilt was used to model the interactions between electrons and ion cores^20^. The Monkhorst–Pack (MP) scheme with a Γ-centered k-point mesh of 15 × 15 × 3 grid is employed to integrate over the first Brillouin zone in the reciprocal space of hexagonal unit cell of Sc_2_SnC^21^. A cutoff energy of 700 eV was chosen to expand the eigenfunctions of the valence and nearly valence electrons using a plane-wave basis. During the geometry optimization, both the total energy and internal forces were minimized with the Broyden-Fletcher-Goldfarb-Shanno (BFGS) algorithm^22^. To achieve the self-consistent convergence the difference in the total energy is kept less than 5 × 10^–6^ eV/atom, the maximum ionic Hellmann–Feynman force less than 0.01 eV/Å, maximum ionic displacement less than 5 × 10^–4^ Å, and maximum stress less than 0.02 GPa.

The elastic properties are investigated using finite-strain theory as embedded in the CASTEP code^23^. In this method, a predetermined value for strain is used to relax all the free parameters and compute the stress. For elastic calculations, the convergence criteria are set as: the difference in total energy less than 10^–6^ eV/atom, the maximum ionic Hellmann–Feynman force less than 2 × 10^–3^ eV/Å, and the maximum ionic displacement less than 10^–4^ Å. The finite-strain theory as implemented in CASTEP has been successfully employed to calculate the elastic properties of numerous systems^24–35^.

Lattice dynamic properties such as phonon dispersion and phonon density of states are calculated using a 3 × 3 × 1 supercell defined by cutoff radius of 5.0 Å employing the finite displacement supercell method within the code. A 35 × 35 × 7 k-point mesh was used to calculate the electronic charge density distribution and the Fermi surface. Defect calculations were carried out with a 3 × 3 × 1 supercell of 72-atomic site (36 M, 18A, and 18 C) under constant pressure. To determine the potential interstitial sites a thorough computational search was performed examining all potential interstitial sites. The calculations with large supercell require comparatively small cutoff energy and moderate k-point mesh. A cutoff energy of 350 eV and a k-point mesh of 3 × 3 × 1 grid in the MP scheme are used for the supercell defect calculations.

## Results and discussions

### Structural properties

**Figure 1:**
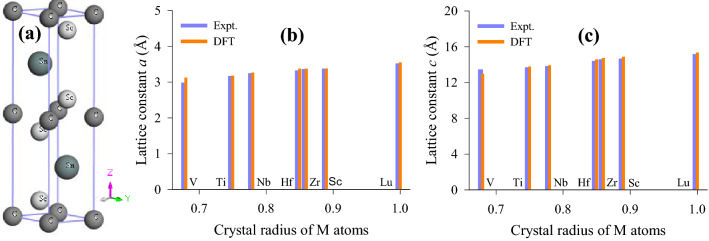
(**a**) Conventional unit cell of Sc_2_SnC as a structural model of 211 M_2_SnC MAX phases; (**b**) lattice constants *a* and (**c**) lattice constants *c* increase with crystal radius of M atoms.

Commonly with other MAX phases Sc_2_SnC crystallizes in the hexagonal space group *P*6_3_/mmc (No. 194)^12^. Each unit cell of Sc_2_SnC contains two formula units and eight atoms (refer to Fig. 1a). The Sc atoms occupy the 4f Wyckoff site with the fractional coordinates (1/3, 2/3, z), the Sn resides in the 2*d* atomic site with the fractional coordinates (1/3, 2/3, 3/4) and the C atoms are accommodated at the 2a Wyckoff position with the fractional coordinates (0, 0, 0). The atomic sites of Sc_2_SnC with theses fractional coordinates are also valid for all 211 MAX phases. The optimized lattice parameters are listed in Table S1 in the supplementary section along with those of all M_2_SnC MAX phases including experimental values^10,12,16,17,36^. The predicted values for Sc_2_SnC are very good agreement with the experimental values, supporting the validity of the present investigation. In previous studies^16,17^, we observed that the lattice constants of Sn-based 211 MAX phases maintain a good relation with the crystal radius of M-atoms. Both lattice constants *a* and *c* increase almost linearly with the crystal radius of M-atoms. The Sn-based new compound Sc_2_SnC also maintains this relationship (see Fig. 1b and c).

### Electronic properties

Electronic band structure, electronic density of states (DOS), charge density map, Fermi surface, Mulliken population analysis are investigated to describe the electronic and bonding features of Sc_2_SnC, a compound in M_2_SnC MAX family.

#### Band structure and DOS

**Figure 2:**
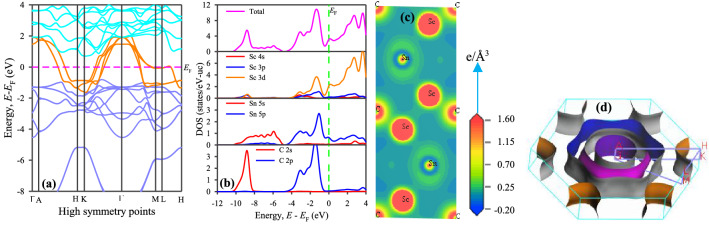
(**a**) Band structure, (**b**) Density of states, (**c**) Charge density map and (**d**) Fermi surface of Sc_2_SnC.

The electronic band structure of Sc_2_SnC was calculated along the high symmetry directions in the first Brillouin zone (refer to Fig. 2a). It reveals the metallic characteristics of Sc_2_SnC, which are similar to other MAX phases including M_2_SnC as the valence band crosses the Fermi level *E*_F_ and overlaps with the conduction band. The Fermi level of Sc_2_SnC intersects the crossing bands roughly along the middle and is located at about equal energies from both the pure valence and conduction bands. Conversely, the Fermi level in V_2_SnC and Ti_2_SnC lies just below the valence band maximum nearby the Γ-point^4,17^. The Γ-point, where most of the valence bands accumulate, shifts upwards for other M_2_SnC MAX phases (see Fig. 6 in Ref. 4). The distance of this point from the Fermi level increases following the order: Ti_2_SnC < V_2_SnC < Nb_2_SnC < Zr_2_SnC < Hf_2_SnC < Sc_2_SnC < Lu_2_SnC. The band features of the Sc_2_SnC are very similar to that of the Lu_2_SnC compared to other M_2_SnC phases^4^. A notable feature in the band structure is the considerable anisotropic nature with low energy dispersion along the c-axis. This is apparent from the reduced dispersion along the short H–K and M–L directions. The anisotropic nature of the band structure near and below the Fermi level indicates that the electrical conductivity is as anisotropic for the Sc_2_SnC as for the other M_2_SnC MAX phases.

To obtain more information on the chemical bonding in Sc_2_SnC, the total and partial densities of states were calculated (refer to Fig. 2b). In this figure, the broken vertical green line refers to the Fermi level *E*_F_, which is located to the left of a pseudogap in the total DOS. It is one of the indications of the structural stability of Sc_2_SnC. The proximity of *E*_F_ to the pseudogap can lead to more structural stability of the compounds of mixed bonding character^37,38^. Comparing the position of *E*_F_ relative to the pseudogap for all existing M_2_SnC MAX phases the structural stability should follow the order: Nb_2_SnC > Ti_2_SnC > Lu_2_SnC > Zr_2_SnC > Hf_2_SnC > Sc_2_SnC > V_2_SnC. The main contribution to the total DOS at *E*_F_ comes from the d-orbital of Sc. The d-resonance at the surroundings of *E*_F_ and the finite value of the total DOS at *E*_F_ indicates the metallic character of Sc_2_SnC and this is a common feature of MAX phases. The total DOS of Sc_2_SnC at *E*_F_ is 3.10 states/eV-uc, which is about half of V_2_SnC (6.12 states/eV-uc) and between the range (2.35–3.93 states/eV-uc) of other M_2_SnC phases^4,17^. Above the *E*_F_, the antibonding states arise due to d-orbitals of M atom in Sc_2_SnC in similar to other M_2_SnC MAX phases.

The valence band of Sc_2_SnC is divided into two main parts. The lower part is situated between −10.4 eV and −4.9 eV, which contains a distinct peak and a flat region. The peak originates as a result of hybridization between Sc 3d and C 2s orbitals, indicating covalent Sc–C bonding. The flat region arises due to the main contribution of Sn 5s electrons. The upper valence band consists of two distinct peaks. The highest peak close to *E*_F_ is also due to the hybridization of Sc 3d electrons with C 2p electrons. Hybridization between Sc 3p and Sn 5p near the *E*_F_ also contributes to the highest peak of the total DOS. This hybridization leads to the formation of the Sc-Sn covalent bond between Sc and Sn. This bond is not as strong as Sc-C because the corresponding peak is closer to the Fermi level. The lowest peak centered at −3.3 eV arises due to the interaction between Sc 3d and C 2p states. The bonding nature of Sc_2_SnC is almost same of other M_2_SnC MAX phases. Overall, the bonding character in Sc_2_SnC is a combination of metallic, covalent, and, owing to the difference in electronegativity between the comprising elements, ionic like other MAX phase compounds.

#### Charge density

The contour map of the electron charge distribution among the constituent atoms in a compound is a way to understand the nature of atomic bonding in the material. The contour map for Sc_2_SnC is given in Fig. 2c. It can be observed that the charge distributions around the atoms have created an almost spherical electron cloud and its intensity determines the amount of charge accumulation. The amount of charge accumulated around the Sc atom is 0.53e, while the amount of charge accumulated around the M atom of other M_2_SnC phases is between 0.28 and 0.45e^4,17^. Clearly, the maximum charge accumulates around the Sc atom among all M-atoms in M_2_SnC phases. The minimum charge accumulates around Hf^4^. The electron cloud of Sc-charge overlaps with that of C-charge and slightly edges with that of Sn-charge, which indicates the stronger covalent Sc–C and weaker covalent Sc–Sn bonding, respectively. The spherical distribution of charge around the atoms is an indication of some ionic character in chemical bonds in Sc_2_SnC. The contour map of electron charge distribution for Sc_2_SnC is almost identical to those of other M_2_SnC phases.

#### Fermi surface

The Fermi surface (FS), one of the most innovative ideas developed by solid-state physicists in the last century, isolates occupied electron states from unoccupied ones at zero temperature. The dynamical properties of an electron on the FS commonly depend on where it is found on the FS, and the shape of the FS in regard to the Brillouin zone can assist as a guide to the electrical properties of a metallic system. Currently, the existence of a FS is likely the utmost significant signature of the entity of Fermi liquid quasiparticles in a material. Indeed, the FS is linked to a variety of fascinating physical phenomena. The FS of Sc_2_SnC was calculated and is shown in Fig. 2d. The FS consists of four Fermi sheets centered along the Γ–A direction. The three Fermi sheets close to the center of the Brillouin zone have cylindrical or prismatic-like hexagonal cross sections. These are 2D-like electron sheets. The remaining sheet consists of six separate parts parallel to the H–K directions. This sheet is hole-like and situated at the corners of the Brillouin zone around the H–K directions. In comparison to the FSs of M_2_SnC family, the FS of Sc_2_SnC is very similar to that of Lu_2_SnC and simple enough compared to the FSs of other M_2_SnC phases^4,17^. The non-spherical shape of the Fermi sheets is an indication of the metallic conductivity of Sc_2_SnC^39^.

#### Mulliken population

Population analysis in CASTEP is carried out using a projection of the planewave (PW) states onto a linear combination of atomic orbitals (LCAO) localized basis using a method developed by Sanchez-Portal et al.^40^. Population analysis of the resultant projected states is then accomplished using the Mulliken formalism^41^. This analysis provides the Mulliken charge, bond population and bond length in a bulk material. Mulliken charge associated with a given atom, A, can be determined as:

1 $$Q\left( A \right) = \mathop \sum \limits_{k} \omega_{k} \sum {\mathop \sum \limits_{\mu }^{on A} \mathop \sum \limits_{v} P_{\mu v} \left( k \right)S_{\mu v} \left( k \right)} \sum$$

where *P*_μν_ (*k*) is the density matrix and *S*_μν_(*k*) is overlap matrix. The bond population between two atoms A and B can be calculated as:

2 $$P\left( {AB} \right) = \mathop \sum \limits_{k} \omega_{k} \sum \mathop \sum \limits_{\mu }^{on A} \mathop \sum \limits_{v}^{on B} 2P_{\mu v} \left( k \right)S_{\mu v} \left( k \right)$$

The Mulliken charge measures the effective valence from the absolute difference between the formal ionic charge and the Mulliken charge on the atomic species. Table S2 lists the effective valence, bond population, and bond length between different atoms in Sc_2_SnC and existing M_2_SnC MAX phases. The pure valence states for transition metals Sc, Ti, V, Zr, Nb, Lu, and Hf in M_2_SnC MAX phases are 3d^1^, 3d^2^, 3d^3^, 4d^2^, 4d^4^ 5s^1^, 5d^1^, 5d^2^, respectively. It is observed that the effective valence largely depends on the d-orbital electrons of the transition metals. It increases when the transition metal moves from the left to the right in the periodic table. Its non-zero positive value is an indication of mixed covalent and ionic nature in chemical bonds. Its progression towards zero value indicates an increase in the level of ionicity. Its zero value implies an ideal ionic character in a chemical bond. Its progression from zero with a positive value indicates an increase in covalency level of chemical bonds. Based on the effective valence the covalency of M_2_SnC increase when M atoms move from the left to the right in the periodic table.

Bond population is another indication of bond covalency in a crystal as a high value of bond population in essence indicates a high degree of covalency in the chemical bond. The M–C bond in the MAX phases is mainly covalent bond. The bond population of M–C bond in each M_2_SnC MAX phases is positive except in Lu_2_SnC. The bond population of M–C bond in M_2_SnC deceases when the M atom moves from the left to the right in the periodic table, indicating the decrease in covalency. Actually, effective valence and positive bond population collectively control the covalency of crystalline solids. The bulk modulus is mostly controlled by the bond covalency. Between effective valence and positive bond population, which is most influential in bond covalency? This can be verified with the bulk modulus. It is observed from the Fig. 4a in the next section that the bulk modulus changes according to the effective valence. Therefore, it can be concluded that the effective valence mainly controls the covalency level in the studied compounds. The bond length of covalent M–C bond deceases when the M atom moves from the left to the right in the periodic table. It is clear that the shorter the covalent bond length, the higher the bond covalency. A negative bond population indicates the antibonding state between two relevant atoms, which weakens the chemical bonding between them. Other bonds in M_2_SnC MAX phases have negative bond population with the exception of the M-Sn bond in Sc_2_SnC and the Sn–C bond in Lu_2_SnC. Indeed, the Sn–C bond is the only source of the covalency in Lu_2_SnC.

### Mechanical properties

Single crystal elastic constants, bulk elastic moduli, elastic anisotropy, Vickers hardness are calculated to describe the mechanical behaviors of Sc_2_SnC in comparison to existing M_2_SnC phases.

#### Single crystal elastic constants

Elastic constants are the fundamental tools for accessing the mechanical behavior of crystalline solids. MAX phases have five independent elastic constants *C*_ij_ due to their hexagonal crystal symmetry. These are *C*_11_, *C*_33_, *C*_44_, *C*_12_ and *C*_13_. In addition they have one more dependent elastic constant *C*_66_, which depends on *C*_11_ and *C*_12_ and *C*_66_ = (*C*_11_–*C*_12_)/2. First of all, the elastic constants justify the mechanical stability of compounds obeying Born criteria. For hexagonal systems these criteria are as follows^42^:

3 $${\text{C}}_{{11}} ,{\text{ C}}_{{33}} ,{\text{ C}}_{{44}} {\text{ > }}0;{\text{ C}}_{{11}} {\text{ > }}\left| {{\text{C}}_{{12}} } \right|{\text{ and }}\;(C_{{11}} + C_{{12}} )C_{{33}} {\text{ > 2C}}_{{13}} {\text{C}}_{{13}}$$

**Figure 3:**
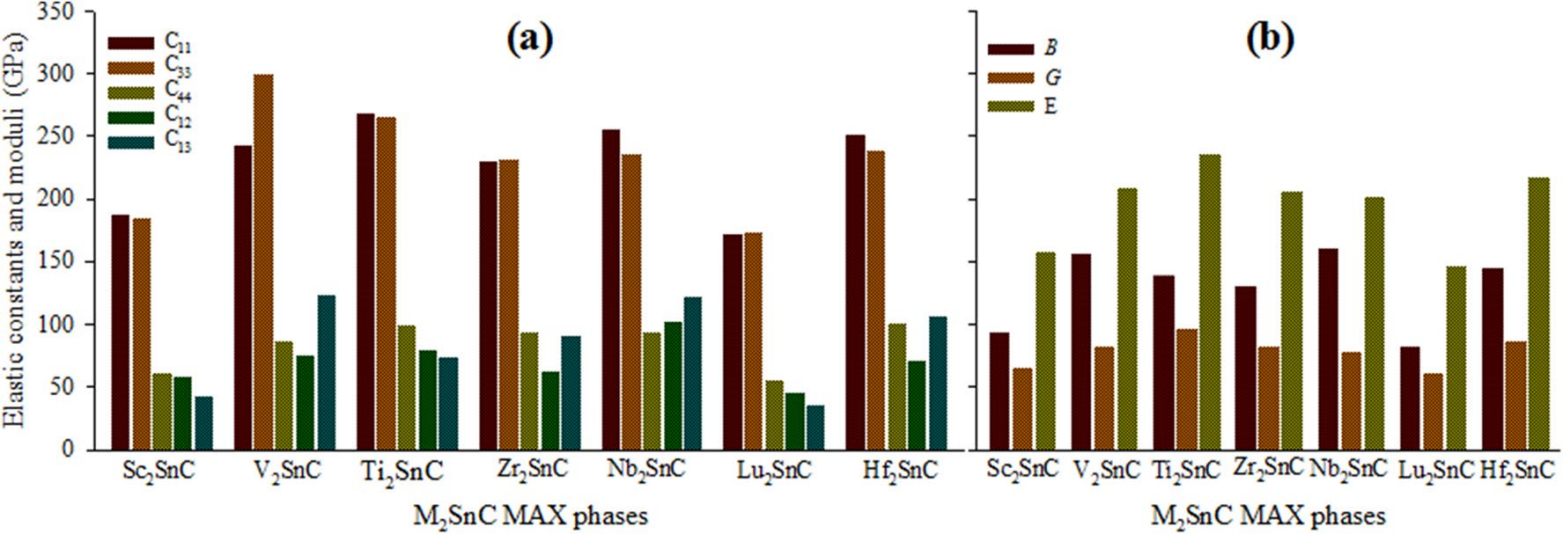
(**a**) Elastic constants *C*_ij_ and (**b**) elastic moduli *B*, *G*, and *E* of Sc_2_SnC and other MAX phases.

The calculated elastic constants of Sc_2_SnC are listed in Table S3 and shown in Fig. 3a along with CASTEP-derived elastic constants for existing M_2_SnC phases for comparison. Sc_2_SnC meets the above conditions by its elastic constants like its predecessors M_2_SnC and ensures its own mechanical stability like its predecessors.

The elastic constants *C*_11_ and *C*_33_ represent the resistance to linear compression, whereas other constants such as *C*_12_, *C*_13_, and *C*_44_ represent the resistance to shape change. Indeed, *C*_11_ and *C*_33_ represent the stiffness along the crystallographic a- and c-axis, respectively. The stiffness of Sc_2_SnC is slightly larger along the a-axis than along the c-axis, which is also observed for Ti_2_SnC, Nb_2_SnC and Hf_2_SnC. For the remaining M_2_SnC phases, V_2_SnC, Zr_2_SnC, and Lu_2_SnC, the stiffness along the c-axis is slightly larger than that in the a-axis. The difference between *C*_11_ and *C*_33_ quantifies the level of elastic anisotropy in crystals relating to the crystallographic axis. Accordingly, V_2_SnC, Nb_2_SnC, and Hf_2_SnC are elastically more anisotropic than Sc_2_SnC, Ti_2_SnC, Zr_2_SnC, and Lu_2_SnC. The new phase Sc_2_SnC ranks fourth in the view of both less and high anisotropy in the M_2_SnC family of seven members.

Shear elastic constants *C*_12_ and *C*_13_ reciprocally lead to a functional stress component along the crystallographic a-axis with a uniaxial strain along the crystallographic b- and c-axis, respectively. This stress component takes the measurements of the shear deformation resistance of a compound along the crystallographic b- and c-axis, when stress is applied along the a-axis. The Nb_2_SnC phase is most capable of resisting such deformation, whereas Lu_2_SnC will easily deform under an equal stress along the a-axis. The new compound Sc_2_SnC will be the second in rank in M_2_SnC systems that will be easily deformed if a rank of deformation resistance of M_2_SnC system is made: Nb_2_SnC > V_2_SnC > Hf_2_SnC > Ti_2_SnC > Zr_2_SnC > Sc_2_SnC > Lu_2_SnC.

The elastic constant *C*_44_ provides an indirect measure of the indentation hardness of a material. A low value of *C*_44_ indicates higher shearability and low hardness of a compound. High shearability and low hardness are related to better machinability of a compound. Due to low value of *C*_44_, Lu_2_SnC has highest shearability among the seven M_2_SnC MAX phases. The new material Sc_2_SnC should be the second in rank in the M_2_SnC systems for shearability.

The symmetry condition *C*_66_ = (*C*_11_–*C*_12_)/2 represents an important consequence in hexagonal crystals. *C*_66_ serves as the shear constant on the (100) plane in a [010] direction, while (*C*_11_–*C*_12_)/2 stands for the shear constant on the (110) plane in a [110] direction. Therefore, the elastic shear constant is the same for all planes in the [001] zone, independent on the specific shear plane or shear direction, which is known as the transverse isotropy. This means that the elastic constants are invariant for arbitrary rotation around the z-axis: in the xy plane, the hexagonal crystals are elastically isotropic, which we will observe in a subsequent section.

#### Bulk elastic moduli

**Figure 4:**
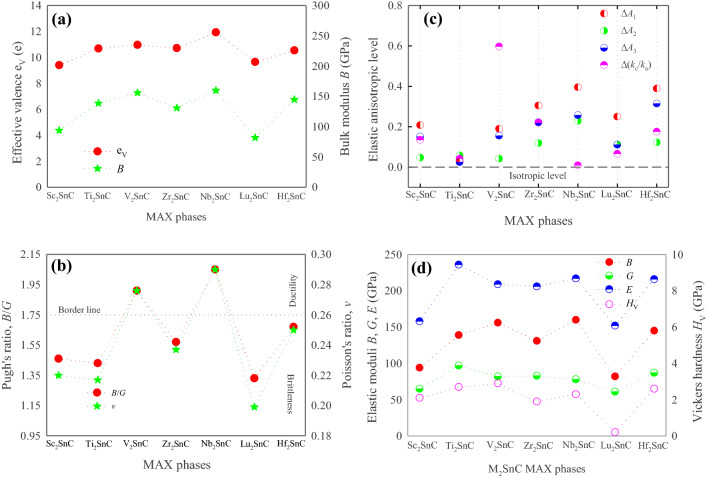
(**a**) Bulk modulus with effective valence; (**b**) Pugh’s and Poisson’s ratio; (**c**) elastic anisotropy level and (**d**) Vickers hardness with elastic moduli of M_2_SnC.

Elastic moduli are the most important elastic parameters that assess the mechanical behavior of crystalline solids. Calculated elastic moduli are listed in Table S3 and shown in Fig. 3b. Bulk modulus *B* and shear modulus *G* can be derived from the elastic constants *C*_ij_ using Voight–Reuss–Hill approximations^43–45^. A detailed discussion of these methods for hexagonal crystals is found in a recent study^46^. The bulk modulus of a crystal depends microscopically on the nature of its bond such as length and type. In the case of the studied compound it is observed that it is also controlled by the total effective valence of the crystal (see Fig. 4a). Bulk modulus is a measure of the resistance to uniform compression of a material and it is linked to chemical composition and crystal structure. Among M_2_SnC phases, the new phase Sc_2_SnC possesses the second lowest value for *B*. The highest value is assigned to Nb_2_SnC and the lowest value is associated with Lu_2_SnC. Therefore, Sc_2_SnC will be compressed more easily as compared to existing M_2_SnC phases except Lu_2_SnC. The shear modulus *G* is concerned with the deformation of a solid material when it experiences a force parallel to one of its surfaces while its opposite face experiences an opposing force such as friction. *G* maintains a good relationship with *C*_44_. Here, it is reflected and Sc_2_SnC secure the second rank in the scale of lowest value of *G* similar to *C*_44_.

*B* and *G* collectively prescribe an important parameter known as Pugh’s ratio (defined as *B*/*G*) that evaluates a necessary mechanical behavior of crystalline solids^47^. Generally, a material is either considered brittle or ductile. The brittle materials have a value less than 1.75 and ductile materials possess a value greater than 1.75. Materials with a *B*/*G* value above or below this borderline value behave in a less ductile or brittle manner. Accordingly, Sc_2_SnC is a brittle material similar to Ti_2_SnC, Zr_2_SnC, Lu_2_SnC and Hf_2_SnC while V_2_SnC and Nb_2_SnC exhibit ductility (refer to Fig. 4b).

Poisson’s ratio *v* is another important parameter and can be derived from *B* and *G*: *v* = (3*B*–2*G*)/(6*B* + 2*G*). Similar to Pugh’s ratio, Poisson’s ratio can serve as a predictor for distinguishing brittle from ductile materials. Poisson’s ratio *v* with a value less than 0.26 identifies the materials as brittle ones and with a higher value the ductile ones^48^. The Poisson’s ratio has classified the M_2_SnC MAX phases into brittle and ductile groups, consistently with Pugh’s ratio above. That is, the Sc_2_SnC can be considered brittle. The Poisson’s ratio can also identify the interatomic forces between atoms in a solid^49^. When the Poisson’s ratio of a solid is between 0.25 and 0.50, the interatomic forces between the atoms of that solid will be the central forces and if the Poisson’s ratio is outside of this range the interatomic forces will be the non-central forces^50^. A central force is a force (possibly negative) that points directly from a particle to a certain point in space, the center and whose magnitude depend only on the distance of the particle from the center while a non-centrifugal force is a force between two particles that is not directed along their connecting line. The Poisson’s ratio of V_2_SnC, Hf_2_SnC and Nb_2_SnC lies in this range and accordingly their interatomic forces are central forces i.e., these compounds are stabilized with the central forces and they are called central-force solids. For central force solids the Cauchy relations are generally established. The interatomic forces in the new phase Sc_2_SnC including remaining M_2_SnC phases are non-central forces as their Poisson’s ratios are outside this range. Consequently, Sc_2_SnC, Zr_2_SnC, Ti_2_SnC and Lu_2_SnC are stabilized with the non-central forces and they are called non-central-force solids. For non-central-force solids the Cauchy relations are generally not established. Moreover, the Poisson’s ratio can predict the bonding nature in solids^51^. A completely covalent crystal is characterized with a Poisson’s ratio equal or less than 0.1. A perfectly metallic compound possesses a Poisson’s ratio equal or greater than 0.33. The Poisson’s ratio of Sc_2_SnC including existing M_2_SnC lies between 0.1 and 0.33, indicating that their chemical bonding is a combination of metallic and covalent natures.

Elastic moduli *B* and *G* also provide another essential property, the Young’s modulus *E* via the relation, *E* = 9*BG*/(3*B* + *G*). The Young’s modulus of a material is a useful property for predicting the behaviour of the material when subjected to a tensile force. Stiffness of a material mostly depends on its Young’s modulus. Higher Young’s modulus is an indication of higher stiffness. In the family of M_2_SnC MAX phases, Ti_2_SnC is the stiffest material and Lu_2_SnC is the softest one. The newly synthesized Sc_2_SnC ranks second on the scale of softness: Lu_2_SnC > Sc_2_SnC > Nb_2_SnC > Zr_2_SnC > V_2_SnC > Hf_2_SnC > Ti_2_SnC. The Young’s modulus of MAX phases can be related to the exfoliation energy. The smaller the Young's modulus, the softer the system and hence the lower the exfoliation energy and the higher the possibility of etching into 2D MXenes^52^. The four MAX phases Ti_2_AlC, Ti_2_AlN, V_2_AlC, and Nb_2_AlC in the 211 family are exfoliated experimentally into MXenes^53^. Their theoretical Young’s moduli^54^ range from 262 to 312 GPa and exfoliation energies^53^ range from 0.164 to 0.205 eV/Å^2^. V_2_AlC has the highest Young’s modulus (~ 312 GPa) and consequently has the highest exfoliation energy (0.205 eV/Å^2^). As the Young’s moduli of the Sn-based 211 MAX phases under study range from 152 to 219 GPa, their exfoliation energies can be expected to be lower than 0.205 eV/Å^2^. Very recently, the exfoliation energies of Sc_2_SnC, Ti_2_SnC, V_2_SnC, Zr_2_SnC, Nb_2_SnC, and Hf_2_SnC are calculated to be 0.131, 0.164, 0.137, 0.157, 0.150, and 0.158 eV/Å^2^, respectively^55^. These values lie within the range between 0.131 and 0.164 eV/Å^2^, which are lower than the range of 0.164 and 0.205 eV/Å^2^. As the Young's modulus of Lu_2_SnC is lowest in the M_2_SnC phases considered here, its exfoliation energy can be expected to lie within this range. The lower the exfoliation energy, the higher the possibility to be etched experimentally into 2D MXenes. Therefore, Lu_2_SnC and other M_2_SnC phases considered here are more likely to be etched into 2D MXenes than V_2_AlC. Further, the Young’s modulus *E* has a good relation to the thermal shock resistance *R*: *R* ∝ 1/E^56^. The lower the Young’s modulus, the better the thermal shock resistance. A material of higher thermal shock resistance (i.e., lower Young’s modulus) has the potential to be used as a TBC material. The Young’s modulus of Sc_2_SnC and other M_2_SnC MAX phases are lower than that of a potential TBC material TiO_2_ whose Young modulus is 283 GPa^57^. Therefore, Sc_2_SnC and other existing M_2_SnC phases have possibility to be TBC materials if they also have high thermal expansion coefficient and melting point, low thermal conductivity, and good oxidation resistance.

#### Elastic anisotropy

The study of elastic anisotropy is important as it influences a variety of physical processes including the development of plastic deformation in crystals, microscale cracking in ceramics, and plastic relaxation in thin-film metallics^58^. For hexagonal crystals like MAX phases the shear anisotropy factors *A*_i_ (i = 1, 2, 3) are studied extensively^16,27,28,31,59^. The equation that determines the shear anisotropy factor *A*_1_, for the {100} shear planes between the < 011 > and < 010 > directions, is *A*_1_ = (*C*_11_ + *C*_12_ + 2*C*_33_4*C*_12_)/6*C*_44_; the equation of *A*_2_, for the {010} shear planes between < 101 > and < 001 > directions, is *A*_2_ = 2*C*_44_/(*C*_11_*C*_12_); and the equation of *A*_3_, for the {001} shear planes between < 110 > and < 010 > directions, is *A*_3_ = (*C*_11_ + *C*_12_ + 2*C*_33_4*C*_13_)/3(*C*_11_*C*_12_). Deviation of *A*_i_ from unity Δ*A*_i_ (= *A*_i_ ~ 1) quantifies the degree of shear anisotropy of crystals. The calculated *A*_i_ is listed in Table S4 and the anisotropy level Δ*A*_i_ is shown in Fig. 4c. Considering the average on all the planes, Ti_2_SnC is elastically less anisotropic and Nb_2_SnC is elastically highly anisotropic. Sc_2_SnC ranks third in view of less anisotropy in the M_2_SnC family: Nb_2_SnC > Hf_2_SnC > Zr_2_SnC > Lu_2_SnC > Sc_2_SnC > V_2_SnC > Ti_2_SnC. Individually, in the {100} shear planes Nb_2_SnC is highly anisotropic; in the {010} shear planes Nb_2_SnC is again highly anisotropic and in the {001} shear planes Hf_2_SnC is highly anisotropic.

The anisotropy level in the hexagonal crystals like MAX phases can also be quantified by another anisotropy factor named compressibility anisotropy factor and it is defined as *k*_c_/*k*_a_ = (*C*_11_ + *C*_12_2*C*_13_)/(*C*_33_*C*_13_)^48^. Here, *k*_a_ and *k*_c_ are the linear compressibility coefficients along the *a*- and c-axis, respectively. Deviation of *k*_c_/*k*_a_ from the unity Δ(*k*_c_/*k*_a_) (= *k*_c_/*k*_a_ ~ 1) quantifies the degree of the compressibility anisotropy of crystals. The calculated *k*_c_/*k*_a_ is listed in Table S4 and Δ(*k*_c_/*k*_a_) is shown in Fig. 4c. The compressibility anisotropy level is highest in V_2_SnC and lowest in Nb_2_SnC. Sc_2_SnC ranks in the middle in the M_2_SnC family of seven members. If *k*_c_/*k*_a_ > 1, the material is more compressible along the c-axis than along the a-axis. Therefore, Sc_2_SnC, Ti_2_SnC and Lu_2_SnC are slightly more compressible along the c-axis than along the a-axis while V_2_SnC, Zr_2_SnC, Nb_2_SnC and Hf_2_SnC are compressed more easily along the a-axis than along the c-axis.

There are some anisotropy factors such as percentage anisotropy factors *A*_B%_ and *A*_G%_ based on the bulk and shear moduli within the Voigt and Reuss limits, which are applicable for all types of crystals. *A*_B%_ measures anisotropy in compression while *A*_G%_ measures anisotropy in shear. These two factors are defined as *A*_B%_ = [(*B*_V_*B*_R_)/(*B*_V_ + *B*_R_)] × 100% and *A*_G%_ = [(*G*_V_*G*_R_)/(*G*_V_ + *G*_R_)] × 100%^51^. The calculated values are listed in Table S4. Both these factors assign zero value for isotropic crystals and their positive values indicate the anisotropy level in crystals. *A*_B%_ is highest for V_2_SnC and lowest for Ti_2_SnC while *A*_G%_ is highest for Hf_2_SnC and lowest for Ti_2_SnC. Sc_2_SnC ranks fourth on the *A*_B%_ scale and second on the *A*_G%_ scale in terms of minimum anisotropy. Universal anisotropy factor *A*^U^ is also applicable for all types of crystals. It is defined as *A*^U^ = 5(*G*_V_/*G*_R_) + (*B*_V_/*B*_R_)6 ≥ 0^17^. Its zero value corresponds to isotropic crystals and a positive value implies the anisotropy level in crystals. The calculated values are listed in Table S4. Hf_2_SnC has the highest value of *A*^U^ and Ti_2_SnC possesses the lowest value. Sc_2_SnC has the second lowest value of *A*^U^.

**Figure 5:**
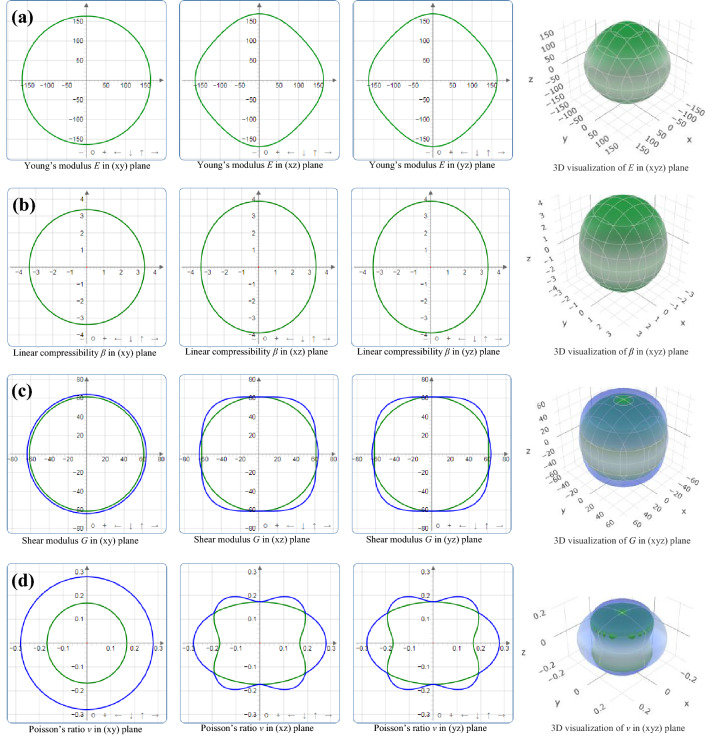
Directional dependence of (**a**) Young’s modulus *E*, (**b**) linear compressibility β, (**c**) shear modulus *G* and (**d**) Poisson’s ratio *ν* of Sc_2_SnC.

The 2D and 3D graphical representations of the directional elastic properties of materials are visualization of elastic anisotropy in crystals. ELATE is an open-source software^60^, which allows the direct visualization of anisotropy level in Young’s modulus (*E*), linear compressibility (β), shear modulus (*G*) and Poisson’s ratio (*v*) on the 3D spherical plot, as well as 2D projections on the (xy), (xz) and (yz) planes. Uniform circular 2D and spherical 3D graphical representations are the indications of isotropic nature of crystals. As the MAX phases are hexagonal crystals, they are elastically isotropic in the xy plane. It is evident that the 2D presentation of *E*, *β*, *G* and *v* of Sc_2_SnC in the xy plane in Fig. 5 are uniformly circular, indicating the isotropic nature of elastic properties of Sc_2_SnC in xy plane. The 2D presentation of *E*, *β*, *G* and *v* of Sc_2_SnC in the xz and yz planes in Fig. 5 is indicating the elastic anisotropy of Sc_2_SnC in those planes. The greater the deviation from the round shape, the higher the anisotropy level in the crystals in that plane.

In 2D and 3D presentations, ELATE uses maximum two colors for *E* and β and maximum three colors for *G* and *v*. The *E* and β are functions of a single unit vector **a**(*θ*, *ϕ*) while *G* and *v* depend on two orthogonal unit vectors **a**(*θ*, *ϕ*) and **b**(*θ*, *ϕ*, *χ*) (**a** in the direction of the stress applied while **b** in the direction of measurement). The spherical coordinates *θ*, *ϕ*, and *χ* can be defined as 0 ⩽ *θ* ⩽ *π*, 0 ⩽ *ϕ* ⩽ 2*π*, and 0 ⩽ *χ* ⩽ 2*π*. Therefore, *E*, β, *G* and *v* can be expressed as *E*(*θ*, *ϕ*), β(*θ*, *ϕ*), *G*(*θ*, *ϕ*, *χ*) and *ν*(*θ*, *ϕ*, *χ*). *G*(*θ*, *ϕ*, *χ*) and *ν*(*θ*, *ϕ*, *χ*) are represented in 3D space via plotting two surfaces *f* and *g* each with the spherical (*θ*, *ϕ*) coordinates. The surfaces *f* and *g* represent the minimal and maximal values over all possible values of *χ*: *f* (*θ*, *ϕ*) = min_*χ*_* X*(*θ*, *ϕ*, *χ*) and *g*(*θ*, *ϕ*) = max_*χ*_* X*(*θ*, *ϕ*, *χ*), respectively. The surface *g* encloses the surface *f*. For this reason, *g* is plotted in translucent blue color in Fig. 5. The surface *f* is represented with solid green color for positive values and translucent red color for negative values. The absence of red color implies that there are no negative values for *E*, β, *G* and *v* of Sc_2_SnC in any direction. The directional dependencies of *E*, β, *G* and *ν* on the xz and yz planes are almost identical for Sc_2_SnC, similar to Nb_2_SnC, Hf_2_SnC and Zr_2_SnC MAX phases^17^. Ti_2_SnC displays almost isotropic nature of *E*, β, *G* and *ν* in the xz and yz planes. The directional dependence of *E*, β, *G*, and *v* in the Lu_2_SnC differs from that of other M_2_SnC phases. For Sc_2_SnC, Ti_2_SnC and Lu_2_SnC, β has almost no directional dependence.

ELATE imparts a quantitative analysis reporting the minimum and maximum values of each modulus along with the directions along which these extrema occur. It also allows determining the directions of special interest for elastic properties, which are not required to be along the crystallographic axes of the material. Additionally, it reports a measurement of the anisotropy *A*_*X*_ of each elastic modulus *X*, which is defined below:

4 $$A_{X} = \left\{ {\begin{array}{*{20}l} {X_{{{\text{max}}}} /X_{{{\text{min}}}} } & {{\text{if sign}}\left( {X_{{{\text{max}}}} } \right) \, = {\text{ sign}}\left( {X_{{{\text{min}}}} } \right)} \\ \infty & {{\text{otherwise}}} \\ \end{array} } \right.$$

The results are listed in Table 1. It is evident that Young’s modulus exhibits maximum anisotropy for Nb_2_SnC and minimum for Ti_2_SnC and Sc_2_SnC ranks second in view of minimum anisotropy. Anisotropy in linear compressibility is maximum for V_2_SnC and minimum for Ti_2_SnC; Sc_2_SnC ranks third in scale of minimum anisotropy. Anisotropy in shear modulus is highest for Hf_2_SnC and lowest for Ti_2_SnC; Sc_2_SnC ranks second in view of minimum anisotropy. Maximum anisotropy of Poisson’s ratio is observed in Hf_2_SnC and minimum in Ti_2_SnC, whereas Sc_2_SnC ranks third in view of minimum anisotropy. The lowest anisotropy is observed for Ti_2_SnC in the M_2_SnC family considering all indicators.

**Table 1:** Minimal and maximal values of each modulus and elastic anisotropy of Sc_2_SnC and existing M_2_SnC^16,17^.

Phases	Young’s modulus (GPa)		Linear compressibility (TPa^–1^)		Shear modulus (GPa)		Poisson’s ratio	
	*E*_min_	*E*_max_	β_min_	β_max_	*G*_min_	*G*_max_	ν_min_	ν_max_
Sc_2_SnC	152.13	168.70	3.383	3.868	61.39	70.89	0.174	0.279
V_2_SnC	188.79	223.85	1.096	2.711	71.36	86.67	0.129	0.388
Hf_2_SnC	168.97	236.44	1.958	2.481	66.85	99.80	0.122	0.390
Lu_2_SnC	143.59	167.39	3.831	3.959	56.84	70.09	0.166	0.264
Nb_2_SnC	168.47	237.13	1.778	2.149	66.30	97.20	0.153	0.417
Ti_2_SnC	233.72	239.43	2.415	2.451	95.41	100.21	0.194	0.225
Zr_2_SnC	174.63	222.77	2.140	2.789	68.42	94.74	0.136	0.338

Elastic anisotropy *A*_*X*_								
	*A*_*E*_		*A*_β_		*A*_*G*_		*A*_ν_	
Sc_2_SnC	1.109		1.143		1.155		1.605	
V_2_SnC	1.186		2.473		1.215		3.022	
Hf_2_SnC	1.399		1.267		1.493		3.195	
Lu_2_SnC	1.166		1.033		1.233		1.589	
Nb_2_SnC	1.408		1.208		1.466		2.730	
Ti_2_SnC	1.024		1.015		1.050		1.159	
Zr_2_SnC	1.276		1.303		1.385		2.483	

#### Theoretical hardness

Hardness is the property of a material that facilitates it to resist plastic deformation, penetration, indentation and scratching. Therefore, hardness is important from an engineering point of view because the resistance to wear by either abrasion or corrosion by steam, oil and water usually increases with hardness. Theoretical modeling for hardness calculation of partially metallic compounds like ternary MAX phases is difficult. Gou et al. developed a model^61^ based on Mulliken bond population^41^ that is able to calculate the theoretical Vickers hardness of MAX phases. According to this model the bond hardness can be calculated as:

5 $$H_{v}^{\mu } = 740 \left( {P^{\mu } - P^{\mu ^{\prime}} } \right)\left( {v_{b}^{\mu } } \right)^{{ - {\raise0.7ex\hbox{$5$} \!\mathord{\left/ {\vphantom {5 3}}\right.\kern-\nulldelimiterspace} \!\lower0.7ex\hbox{$3$}}}}$$

where ${P}^{\mu }$ denotes to the positive Mulliken bond overlap population of the *μ*-type bond, $P^{\mu ^{\prime}}$ represents the metallic population that is derived from the unit cell volume *V* and the number of free electrons in the cell, *n*_free_ with the formula, $P^{\mu ^{\prime}} = \frac{{n_{free} }}{V},$ here ${n}_{free}= {\int }_{{E}_{P}}^{{E}_{F}}N\left(E\right)dE$ and *E*_P_ and *E*_F_ define the energies at the pseudogap and at the Fermi level, respectively, and ${v}_{b}^{\mu }$ is the bond volume of a *μ*-type bond calculated using the equation ${v}_{b}^{\mu }= {\left({d}^{\mu }\right)}^{3}/\sum_{\mu }\left[{\left({d}^{\mu }\right)}^{3} {N}_{b}^{\mu }\right],$ here ${d}^{\mu }$ and ${N}_{b}^{\mu }$ are respectively the bond length and bond number of *μ* type bonds per unit volume.

When a compound has a positive bond population for multiple bonds, the following equation is used to calculate its Vickers hardness:

6 $$H_{V} = [\mathop \Pi \limits^{\mu } (H_{v}^{\mu } )^{{n^{\mu } }} ]^{{1/\Sigma n^{\mu } }}$$

where *n*^*µ*^ represents the number of *μ*-type bonds. Table S5 lists the Vickers hardness of Sc_2_SnC and other M_2_SnC MAX phases. Sc_2_SnC is harder than Lu_2_SnC and Zr_2_SnC and softer than Ti_2_SnC, V_2_SnC, Nb_2_SnC and Hf_2_SnC. We have found two sets of experimental Vickers hardness for Ti_2_SnC, Zr_2_SnC, Hf_2_SnC, and Nb_2_SnC^36,62^. Experimental values show deviations from one set to another except for Ti_2_SnC. This can be due to sample purity and errors induced by the instruments. Furthermore, the experiment is performed with a sample that contains grains. The grain size largely controls the plasticity as well as the hardness of the compounds. The experimental temperature may be another reason. Although the theoretical values deviate from the experimental values they remain within the typical values (2–8 GPa) for MAX phases. Lu_2_SnC has a low value for *H*_V_, which is very small compared to the lower limit (2 GPa) for the MAX phases. The reason may be the absence of covalent M–C bonds in Lu_2_SnC. Indeed, all M_2_SnC phases have low hardness compared to most of the MAX phases. Consequently, M_2_SnC phases are soft compared to other MAX phases.

In general, the hardness of a compound has a better relationship with its shear and Young’s modulus than bulk modulus^3^. We have plotted the Vickers hardness of M_2_SnC MAX phases in Fig. 4d along with their elastic moduli. For the M_2_SnC MAX phases, it is observed that the hardness follows the trend of bulk modulus instead of shear and Young’s modulus. Further verification is needed to determine whether this trend continues for the carbide MAX phase with a specific A-group element.

### Lattice dynamics

The subject of lattice dynamics is the study of the vibrations of the atoms in a crystal. The vibrations of the atoms are related to many important physical properties such as lattice thermal conductivity, minimum thermal conductivity, Debye temperature, melting point, phonon dispersion, phonon DOS etc. These properties are investigated for newly synthesized Sc_2_SnC to compare with existing M_2_SnC phases.

#### Debye temperature

Debye temperature is a characteristic temperature at which the highest-frequency mode (and hence every possible mode) is excited. It is related to many physical properties such as thermal expansion, thermal conductivity, specific heat and lattice enthalpy. The Anderson method is simple and rigorous way to calculate the Debye temperature of crystalline materials using the equation^63^:

7 $$\theta_{{\text{D}}} = \frac{h}{{k_{{\text{B}}} }}\left[ {\left( {\frac{3n}{{4\pi }}} \right)\frac{{N_{{\text{A}}} \rho }}{M}} \right]^{1/3} v_{{\text{m}}}$$

All symbols bear the conventional meanings and *v*_m_ refers to the average sound velocity, which can be determined using the following equation:

8 $$v_{{\text{m}}} = \left[ {\frac{1}{3}\left( {\frac{1}{{v_{{\text{l}}}^{3} }} + \frac{2}{{v_{{\text{t}}}^{3} }}} \right)} \right]^{ - 1/3}$$

Here, *v*_l_ and *v*_t_ are the longitudinal and transverse sound velocities, respectively. They can be calculated from the bulk and shear moduli *B* and *G* using the equations:

9 $$v_{{\text{l}}} = \left( {\frac{3B + 4G}{{3\rho }}} \right)^{1/2} \;and\;v_{{\text{t}}} = \left( {\frac{G}{\rho }} \right)^{1/2}$$

**Figure 6:**
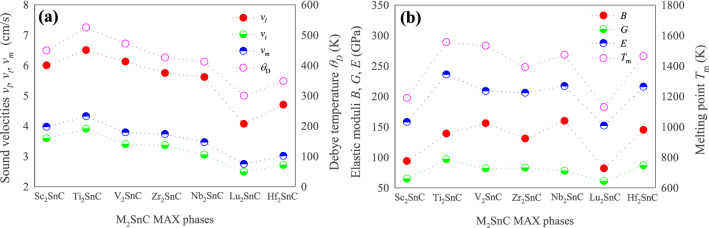
(**a**) Debye temperature with sound velocities; (**b**) melting temperature with elastic moduli.

The calculated values of *θ*_D_ for M_2_SnC MAX phases are listed in Table S6 along with the relevant quantities. The Debye temperature of Sc_2_SnC is the third highest in the M_2_SnC family. In this family, Ti_2_SnC possesses the highest Debye temperature and Lu_2_SnC has the lowest Debye temperature. The Debye temperature of M_2_SnC phases largely depends on the sound velocities and follows the trend of change of sound velocities with the transition metal M (refer to Fig. 6a).

#### Melting point

Melting point of hexagonal crystals like MAX phases can be calculated from elastic constants using: *T*_m_ = 354 + 1.5(2*C*_11_ + *C*_33_)^64^. The calculated values are listed in Table S6. The new phase Sc_2_SnC possesses the second lowest melting point. The highest melting point is obtained for Ti_2_SnC and the lowest melting point is observed for Lu_2_SnC. A higher melting point indicates greater interatomic forces in crystals. Interatomic forces mainly control the bulk elastic properties of crystalline solids. Thus, a relationship can exist between the elastic modulus and the melting temperature of the crystals. Considering this we plotted the elastic moduli and the melting point of M_2_SnC MAX phases in Fig. 6b. It is observed that the melting point has better correlation with bulk modulus *B* and Young’s modulus *E* than shear modulus *G*.

#### Lattice thermal conductivity

The lattice thermal conductivity arises from contributions of phonons of all frequencies. The knowledge of lattice thermal conductivity is important to determine the applicability of a material for use in high temperature environments. Recently, room temperature lattice thermal conductivity of two MAX phases Zr_2_SeC and Zr_2_SC are reported, which are 75 and 80.7% of the total thermal conductivity of the compounds, respectively^11^. So, the lattice thermal conductivity of metallic compounds like MAX phases provides the concept of total thermal conductivity in a computationally tractable way. For this reason, many authors have reported lattice thermal conductivity of many compounds including MAX phases^11,65–68^ Encouraged by these reports we have calculated the lattice thermal conductivity of the newly synthesized Sc_2_SnC MAX phase. Here, the Slack model is used to calculate the lattice thermal conductivity of Sc_2_SnC as MAX phases have dual characteristics of metals and ceramics^69^. The following equation is used in this model:

10 $$k_{{{\text{ph}}}} = A\frac{{M_{{{\text{av}}}} \theta_{D}^{3} \delta }}{{\gamma^{2} n^{2/3} T}}$$

**Figure 7:**
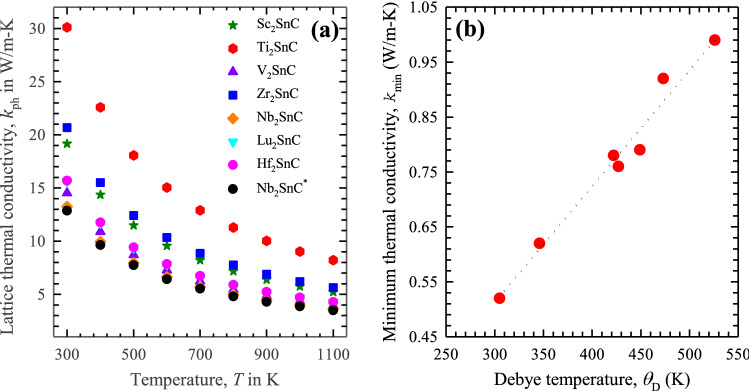
(**a**) Lattice thermal conductivity as a function of temperature. Theoretical data for Nb_2_SnC are taken from Ref. 59^*^; (**b**) minimum thermal conductivity as a function of Debye temperature.

The details of this model are given in a recent study^70^. The room temperature lattice thermal conductivity of Sc_2_SnC and other M_2_SnC phases are listed in Table S6 and their temperature dependency is shown in Fig. 7a. The Debye temperature of the M_2_SnC phases is calculated to range from 300 to 525 K. This implies that all vibrational modes will be active above these temperatures for the M_2_SnC MAX phases. Consequently, from these temperatures and above the phonon contribution becomes dominant in the total thermal conductivity. Here, we report the lattice thermal conductivity of M_2_SnC MAX phases in the temperature range of 300 to 1100 K. In this temperature range, the electronic contribution to the total thermal conductivity should be insignificant. The room temperature (300 K) thermal conductivity of 60 W K^–1^ m^–1^, measured in Ti_2_SC is the highest to date, despite the fact that its electrical conductivity (1.926 × 10^6^ Ω^–1^ m^–1^) is relatively poor. This is due to the large contribution from phonons^71^. For the temperatures at and above 300 K, the lattice contribution should dominate the total thermal conductivity of MAX phases. For comparison, we have the literature values for lattice thermal conductivity of Nb_2_SnC^65^, which are also plotted in Fig. 7. The present values show good agreement with the literature values. It is observed that Sc_2_SnC has the third highest lattice thermal conductivity in the entire temperature range and Ti_2_SnC and Nb_2_SnC possess the highest and lowest values, respectively. The lattice thermal conductivity of M_2_SnC MAX phases decrease gradually with the increase of temperature. The rate of decrease is almost similar for all M_2_SnC phase. Sc_2_SnC should be suitable candidate as a TBC material as other M_2_SnC phases are^17^.

#### Minimum thermal conductivity

Thermal conductivity decreases with increasing temperature. Thus, the minimum value of thermal conductivity is significant for the application of materials at high temperature conditions, for instance, materials selection and design for thermoelectric, thermal barrier coating and other thermal management applications. The concept of a minimum thermal conductivity, *κ*_min_, carried by the atomic vibrations of any solid material led to the development of different models. The Clarke model has become popular for determining the minimum thermal conductivity of solids via the Eq.^72^:

12 $$\kappa_{\min } = { }k_{{\text{B}}} v_{{\text{m}}} \left( {\frac{{nN_{{\text{A}}} \rho }}{M}} \right)^{2/3}$$

All symbols in Eq. (12) are consistent to the symbols used in Eq. (7). The calculated value of *κ*_min_ is listed in Table S6. The phase Sc_2_SnC has the third highest value for *κ*_min_. The highest value is found for V_2_SnC and the lowest value is observed for Lu_2_SnC. The ultralow minimum thermal conductivity of 1.25 W/m–K is used for screening the appropriate materials for TBC application^73^. The values of *κ*_min_ for M_2_SnC MAX phases are lower than this optimum value. Therefore, all M_2_SnC phases should be promising TBC materials with greater possibility for Lu_2_SnC. The minimum thermal conductivity has a linear correlation with the Debye temperature for M_2_SnC phases (Fig. 7b).

#### Phonon dispersion and phonon DOS

**Figure 8:**
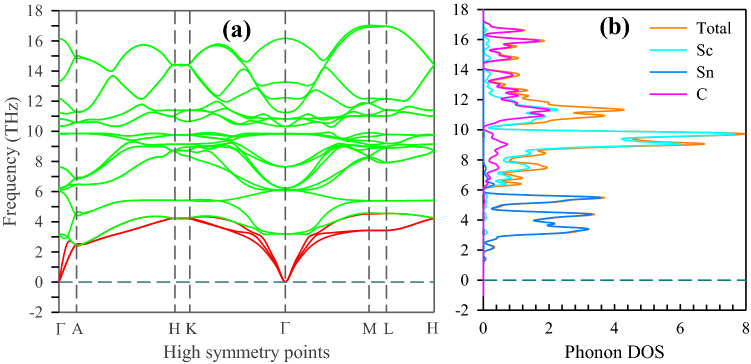
(**a**) Phonon dispersion and (**b**) Phonon DOS.

It is important to study the phonon dispersion and phonon density of states (DOS) to verify the dynamical stability of the crystalline solids. The calculated phonon dispersion is shown in Fig. 8a. For a dynamically stable crystal, there are always three phonons with zero frequency at Γ-point, which corresponds to *k* = 0 in reciprocal space. The phonon branches starting at ω(*k*) = 0 are called acoustic phonon dispersion curves^74^. In the case of Sc_2_SnC (refer to Fig. 8a) the acoustic branches start at ω(*k*) = 0 and consequently indicate the dynamical stability of Sc_2_SnC. The phonons, whose frequencies are non-zero at the Γ point, are called optical phonons. In a number of high-symmetry crystals, and along the high-symmetry directions, the atomic vibrations are either polarized along the propagation wave vector *k*, or perpendicular to *k*. Acoustic modes have one longitudinal acoustic (LA) mode, and two transverse acoustic (TA) modes. A crystal consisting of a unit cell of *N*-atoms has 3*N*–3 optical modes. Accordingly, 211 MAX phases have 21 optical modes. In the Fig. 8a, the acoustic branches are shown with red and optical branches are identified with green.

The calculated phonon DOS of Sc_2_SnC is shown in Fig. 8b, revealing that the acoustic and the lower optical modes arise due to the vibration of heavier Sn-atoms. The middle optical branches arise due to the vibration of Sc-atoms. The higher optical branches mostly originate from the vibration of lighter C-atoms. Acoustic phonon is caused by the coherent vibrations of atoms in a lattice outside their equilibrium position. Conversely, the optical phonon originates due to the out-of-phase oscillation of the atom in the lattice when an atom moves to the left and its neighbor to the right. Most of the optical properties of the crystals are controlled by the optical phonons.

Zone-center phonon modes are of particular interest in the lattice dynamics of crystal solids. Since the Sn-based 211 MAX phases consist of 8 atoms, they have 24 phonon branches or vibration modes. Three of these are acoustic modes with zero frequency at Γ-point and the remaining 21 are optical modes. Of these 21 optical modes, six are IR active, seven are Raman active and the remaining eight are silent modes. Consistent with the factor group theory, the irreducible representations of the Brillouin zone-center optical phonon modes can be classified as:

$$\Gamma_{{{\text{opt}}.}} = {\text{ 2A}}_{{{\text{2u}}}} + {\text{ 4E}}_{{{\text{1u}}}} + {\text{ 2E}}_{{{\text{1g}}}} + {\text{ 4E}}_{{{\text{2g}}}} + {\text{ A}}_{{{\text{1g}}}} + {\text{ 2B}}_{{{\text{1u}}}} + {\text{ 2B}}_{{{\text{2g}}}} + {\text{ 4E}}_{{{\text{2u}}}}$$

where A_2u_ and E_1u_ are IR active and A_1g_, E_1g_ and E_2g_ are Raman active and B_1u_, B_2g_ and E_2u_ are silent modes. The total modes obtained for the M_2_SnC phases in this study are consistent with previous theoretical studies of the lattice dynamics of the various 211 MAX phases^75–77^. Each mode has a specific frequency of vibration. Sometimes two or more modes have the same frequency but it cannot be claimed that they are distinct modes; these modes are called degenerate. For this reason, Table 2 contains six IR active modes and seven Raman active modes. The highest frequencies of the IR active modes are observed at 442.5, 546.3, 621.9, 503.5, 576.0, 459.8 and 600.2 cm^–1^ for Sc_2_SnC, Ti_2_SnC, V_2_SnC, Zr_2_SnC, Nb_2_SnC, Lu_2_SnC and Hf_2_SnC, respectively, while the highest Raman active modes for these compounds are observed at 407.7, 372.1, 274.7, 224.3, 275.0, 171.8 and 179.5 cm^–1^, respectively.

**Table 2:** Theoretical wavenumbers *ω* and symmetry assignment of the IR-active and Raman-active modes of the Sn-based M_2_SnC MAX phases.

Mode		Irr. Rep	Wavenumbers *ω* (cm^1^)						
			Sc_2_SnC	Ti_2_SnC	V_2_SnC	Zr_2_SnC	Nb_2_SnC	Lu_2_SnC	Hf_2_SnC
IR	*ω*_1_	A_2u_	201.9	263.0	110.0	197.2	198.4	145.2	186.0
	*ω*_2_	E_1u_	207.5	223.6	105.1	119.0	127.8	75.7	87.7
	*ω*_3_	E_1u_	344.8	535.7	621.9	495.3	576.0	339.9	600.2
	*ω*_4_	A_2u_	442.5	546.3	560.1	503.5	537.6	459.8	558.0
Raman	*ω*_1_	E_2g_	106.1	65.4	96.2	67.4	65.8	68.7	73.8
	*ω*_2_	E_2g_	328.0	365.8	257.6	192.7	211.6	110.2	133.8
	*ω*_3_	E_1g_	328.6	372.1	260.8	202.2	220.9	109.7	138.5
	*ω*_4_	A_1g_	407.7	347.7	274.7	224.3	275.0	171.8	179.5

#### Defect processes

The motivation to examine the point defect processes of materials stems from the fact that they can impact the macroscopic materials properties (i.e. radiation tolerance)^78–80^. In that respect the investigation of point defects in MAX phases is very important as they can be in radiation environments given that they are considered for nuclear applications^81–83^.

Table 3 lists the defect reactions and the corresponding defect energies for Sc_2_SnC and the existing M_2_SnC MAX phases. In these calculations we have considered all the possible point defects including all the interstitial sites existing in the 211 M_2_SnC MAX phases. The preferable sites are shown in Fig. S1. For the defect reactions we employed the Kröger-Vink notation^84^. In this notation M_i_ stands for an M interstitial defect, V́_Sn_ for a Sn vacant site and M_Sn_ an M atom residing in a Sn site (known as antisite defect). Typically, the energies of the Schottky reaction in this system are high (Table 3) and therefore the Frenkel reactions (Table 3, relations 1–3) or the antisite reactions (Table 3, relations 4–6) are more relevant when considering the radiation tolerance of the material. For Sc_2_SnC the C-Frenkel energy is only 3.33 eV inferring that this is not a particularly radiation tolerant MAX phase as compared to most of the other MAX phases considered here (Table 3). Commonly, with the other MAX phases in Table 3 there is the possibility to form antisite vacancies via the recombination of self-interstitials and vacancies. For Sc_2_SnC this is inferred by the negative energies in reactions 7–9 and 12.

**Table 3:** The defect reaction energies as calculated for Sc_2_SnC and existing M_2_SnC^4,17^MAX phases.

	Reaction (V́ denotes vacancy)	Defect energy (eV)						
		Sc_2_SnC	V_2_SnC	Lu_2_SnC	Ti_2_SnC	Zr_2_SnC	Hf_2_SnC	Nb_2_SnC
1	M_M_ → V́_M_ + M_i_	7.35	6.40	6.61	8.75	8.66	9.34	8.70
2	Sn_Sn_ → V́_Sn_ + Sn_i_	4.21	7.95	3.57	8.97	6.63	7.51	7.56
3	C_C_ → V́_C_ + C_i_	3.33	5.12	2.23	6.10	5.34	4.68	5.18
4	M_M_ + Sn_Sn_ → M_Sn_ + Sn_M_	3.98	4.67	3.67	4.92	4.83	4.72	5.12
5	M_M_ + C_C_ → M_C_ + C_M_	11.31	9.37	11.79	12.81	15.40	16.37	12.64
6	Sn_Sn_ + C_C_ → Sn_C_ + C_Sn_	8.52	8.64	7.75	9.98	9.64	10.07	10.05
7	Sn_i_ + V́_M_ → Sn_M_	− 4.57	− 5.17	− 3.61	− 6.86	− 4.71	− 5.17	− 4.34
8	C_i_ + V́_M_ → C_M_	− 1.65	− 0.80	− 0.13	− 1.07	0.12	1.47	− 0.48
9	M_i_ + V́_Sn_ → M_Sn_	− 3.01	− 4.51	− 2.90	− 5.94	− 5.75	− 6.96	− 6.79
10	C_i_ + V́_Sn_ → C_Sn_	1.26	0.03	1.56	− 0.19	0.22	0.89	− 0.10
11	M_i_ + V́_C_ → M_C_	2.27	− 1.35	3.08	− 0.97	1.28	0.88	− 0.76
12	Sn_i_ + V́_C_ → Sn_C_	− 0.29	− 4.46	0.39	− 4.91	− 2.55	− 3.01	− 2.58
13	M_i_ + Sn_Sn_ → M_Sn_ + Sn_i_	1.20	3.44	0.67	3.03	0.88	0.55	0.76
14	M_i_ + C_C_ → M_C_ + C_i_	5.61	3.77	5.31	5.13	6.62	5.56	4.42
15	Sn_i_ + M_M_ → Sn_M_ + M_i_	2.78	1.24	3.01	1.89	3.95	4.17	4.36
16	Sn_i_ + C_C_ → Sn_C_ + C_i_	3.04	0.66	2.62	1.19	2.79	1.67	2.60
17	C_i_ + M_M_ → C_M_ + M_i_	5.70	5.60	6.49	7.69	8.78	10.81	8.22
18	C_i_ + Sn_Sn_ → C_Sn_ + Sn_i_	5.47	7.98	5.13	8.79	6.85	8.40	7.46
	Schottky reaction	9.78	5.83	9.99	7.97	9.69	8.57	6.70

## Conclusions

In summary, we have employed DFT calculations to investigate the structural, electronic, mechanical and lattice dynamical properties of Sc_2_SnC including defect processes to compare with those of existing M_2_SnC MAX phases. The calculated structural properties show fair agreement with the available experimental values. The structural, mechanical and dynamical stability of Sc_2_SnC is verified. The chemical bonding of Sc_2_SnC is a combination of metallic, covalent and ionic. The softness, elastic anisotropy level and deformability of Sc_2_SnC are moderate compared to the other M_2_SnC phases. Sc_2_SnC has the potential to be etched into 2D MXenes and be a promising TBC material, similar to the other M_2_SnC phases. The hardness of M_2_SnC, including Sc_2_SnC, follows the trend of bulk modulus rather than shear and Young’s modulus while the melting point has a better relationship with the bulk and Young’s modulus than with the shear modulus. The rate of declination of lattice thermal conductivity with temperature is almost similar for all M_2_SnC phases. The minimum thermal conductivity shows a linear relationship with the Debye temperature. The highest frequency of the IR active modes for Sc_2_SnC is lowest in the M_2_SnC family while the highest frequency of the Raman active mode is largest for Sc_2_SnC. Examining the defect processes of the existing M_2_SnC phases it is revealed that Sc_2_SnC is less radiation tolerant than numerous 211 MAX phases.

## Supplementary Information

Supplementary Information.

## Data Availability

All data generated or analysed during this study are included in this published article and its supplementary information files.
