# Supplementary material for: DFT insights into the electronic structure, mechanical behaviour, lattice dynamics and defect processes in the first Sc-based MAX phase Sc2SnC
Source: Sci Rep. 2022 Aug 18;12:14037. doi: 10.1038/s41598-022-18336-z (PMC9388654; doi:10.1038/s41598-022-18336-z)
Supplement: Supplementary file 1 — Supplementary Information. [file 41598_2022_18336_MOESM1_ESM.docx]

Supplementary Information

DFT insights into the electronic structure, mechanical behaviour, lattice dynamics and defect processes in the first Sc-based MAX phase Sc_2_SnC

**M.A. Hadi^1*^, S.-R.G. Christopoulos^2^, A. Chroneos^3,4^, S.H. Naqib^1^, A.K.M.A. Islam^1,5^**

^1^Department of Physics, University of Rajshahi, Rajshahi 6205, Bangladesh

^2^Faculty of Engineering, Environment and Computing, Coventry University, Priory Street, Coventry CV1 5FB, UK

^3^Department of Electrical and Computer Engineering, University of Thessaly, 38221 Volos, Greece

^4^Department of Materials, Imperial College, London SW7 2AZ, UK

^5^International Islamic University Chittagong, Kumira, Chittagong-4318, Bangladesh

**Correspondence:* [*hadipab@gmail.com*](mailto:hadipab@gmail.com)

**Table** **S1**. Lattice parameters (*a*, *c*, *z*_M_ in Å), hexagonal ratio (*c*/*a*) and cell volume (*V* in Å^3^) of Sc_2_SnC and existing M_2_SnC MAX phases.

| Compound | *a* | *c* | *c*/*a* | *z*_M_ | *V* | Remarks |
| --- | --- | --- | --- | --- | --- | --- |
| Sc_2_SnC | 3.373 | 14.854 | 4.403 | 0.5814 | 146.4 | Calc.This |
|  | 3.368 | 14.653 | 4.351 | 0.5786 | 143.9 | Expt.^1^ |
| V_2_SnC | 3.121 | 12.947 | 4.148 | 0.0759 | 109.2 | Calc.^2^ |
|  | 2.9792 | 13.4441 | 4.513 | 0.0744 | 103.3 | Expt.^3^ |
| Ti_2_SnC | 3.172 | 13.772 | 4.342 | 0.0806 | 120.0 | Calc.^4^ |
|  | 3.1635 | 13.675 | 4.323 | --------- | 118.5 | Expt.^5^ |
| Nb_2_SnC | 3.258 | 13.918 | 4.272 | 0.0820 | 128.0 | Calc.^4^ |
|  | 3.2408 | 13.802 | 4.259 | --------- | 125.5 | Expt.^5^ |
| Hf_2_SnC | 3.367 | 14.548 | 4.320 | 0.0865 | 142.9 | Calc.^4^ |
|  | 3.3199 | 14.388 | 4.334 | --------- | 137.3 | Expt.^5^ |
| Zr_2_SnC | 3.367 | 14.730 | 4.374 | 0.0849 | 144.7 | Calc.^4^ |
|  | 3.3576 | 14.568 | 4.339 | --------- | 142.2 | Expt.^5^ |
| Lu_2_SnC | 3.546 | 15.323 | 4.320 | 0.0850 | 166.9 | Calc.^4^ |
|  | 3.514 | 15.159 | 4.314 | --------- | 162.1 | Expt.^5^ |

**Table S2**. Effective valence charge, bond population and bond length of M_2_SnC MAX phases

|  | Sc_2_SnC | Ti_2_SnC | V_2_SnC | Zr_2_SnC | Nb_2_SnC | Lu_2_SnC | Hf_2_SnC |
| --- | --- | --- | --- | --- | --- | --- | --- |
| Atom | Effective valence charge (e) | | | | | | |
| M | 2.47 | 3.58 | 3.68 | 3.57 | 4.69 | 2.55 | 3.72 |
| Sn | 3.79 | 3.89 | 3.99 | 3.93 | 3.93 | 3.54 | 3.69 |
| C | 3.15 | 3.22 | 3.36 | 3.22 | 3.32 | 3.57 | 3.14 |
| Bond | Bond population | | | | | | |
| M-C | 1.17 | 1.08 | 1.02 | 1.05 | 0.99 | –6.25 | 1.39 |
| M-M | –0.52 | –0.34 | –0.42 | –0.09 | –0.26 | –3.62 | –0.31 |
| M-Sn | 0.14 | –0.35 | –0.35 | –0.66 | –1.03 | –1.16 | –0.16 |
| Sn-C | –0.13 | –0.13 | –0.18 | –0.05 | –0.05 | 0.12 | –0.19 |
| Bond | Bond length (Å) | | | | | | |
| M-C | 2.293 | 2.141 | 2.053 | 2.312 | 2.200 | 2.428 | 2.316 |
| M-M | 3.106 | 2.878 | 2.667 | 3.168 | 2.958 | 3.314 | 3.180 |
| M-Sn | 3.172 | 2.966 | 2.886 | 3.113 | 3.001 | 3.255 | 3.071 |
| Sn-C | 4.193 | 3.900 | 3.705 | 4.163 | 3.955 | 4.345 | 4.123 |

**Table S3**. Elastic constants *C*_ij_ and moduli *B*, *G*, *E* in GPa and Poisson’s and Pugh’s ratio *v* and *B*/*G* of Sc_2_SnC including M_2_SnC^2,4^.

| Phases | *C*_11_ | *C*_33_ | *C*_44_ | *C*_66_ | *C*_12_ | *C*_13_ | *B* | *G* | *E* | *v* | *B*/*G* |
| --- | --- | --- | --- | --- | --- | --- | --- | --- | --- | --- | --- |
| Sc_2_SnC | 188 | 186 | 61 | 64 | 60 | 44 | 95 | 65 | 158 | 0.223 | 1.471 |
| Ti_2_SnC | 266 | 266 | 100 | 95 | 75 | 72 | 137 | 98 | 237 | 0.213 | 1.406 |
| V_2_SnC | 246 | 264 | 85 | 84 | 79 | 99 | 145 | 83 | 209 | 0.261 | 1.758 |
| Zr_2_SnC | 228 | 231 | 95 | 83 | 62 | 91 | 130 | 83 | 206 | 0.236 | 1.560 |
| Nb_2_SnC | 267 | 257 | 97 | 87 | 93 | 126 | 165 | 85 | 217 | 0.280 | 1.943 |
| Lu_2_SnC | 176 | 180 | 57 | 65 | 46 | 37 | 86 | 63 | 152 | 0.206 | 1.370 |
| Hf_2_SnC | 247 | 240 | 100 | 88 | 72 | 107 | 145 | 86 | 216 | 0.252 | 1.681 |

**Table S4**. Elastic anisotropy factors of Sc3SnC including existing M_2_SnC^2,4^ MAX phases.

| Phases | *A*_1_ | *A*_2_ | *A*_3_ | *k*_c_/*k*_a_ | *A_B%_* | *A_G%_* | *A^U^* |
| --- | --- | --- | --- | --- | --- | --- | --- |
| Sc_2_SnC | 1.2184 | 0.9534 | 1.1616 | 1.1199 | 0.0769 | 0.2773 | 0.0293 |
| Ti_2_SnC | 0.9703 | 1.0504 | 1.0191 | 1.0147 | 0.0011 | 0.0246 | 0.0025 |
| V_2_SnC | 0.8863 | 1.0200 | 0.9041 | 0.7696 | 0.2402 | 0.1546 | 0.0203 |
| Zr_2_SnC | 0.6855 | 1.1418 | 0.7827 | 0.7675 | 0.2353 | 1.0100 | 0.1067 |
| Nb_2_SnC | 0.6333 | 1.1179 | 0.7079 | 0.8276 | 0.0926 | 1.4360 | 0.1475 |
| Lu_2_SnC | 1.2679 | 0.8774 | 1.1124 | 1.0335 | 0.0068 | 0.4086 | 0.0412 |
| Hf_2_SnC | 0.6226 | 1.1380 | 0.7085 | 0.7890 | 0.1637 | 1.5501 | 0.1607 |

**Table** **S5.** Bond number *n^μ^*, bond length, *d*^μ^ (Å), bond population *P*^μ^, bond volume $v_{b}^{\mu}$ (Å^3^), bond hardness $H_{\nu}^{\mu}$ (GPa), metallic population *P*^μ′^, and hardness $H_{V}$ (GPa) of Sc_2_SnC and existing M_2_SnC MAX phases.

| Compound | Bond | *n*^μ^ | *d*^μ^ | *P*^μ^ | *P*^μ′^ | $v_{b}^{\mu}$ | $H_{\nu}^{\mu}$ | *H*_V_ | *H*_V_ (expt.) |
| --- | --- | --- | --- | --- | --- | --- | --- | --- | --- |
| Sc_2_SnC | Sc-C | 4 | 2.2927 | 1.17 | 0.00568 | 36.60 | 2.1 | 2.1 |  |
| Ti_2_SnC | Ti–C | 4 | 2.1414 | 1.08 | 0.01525 | 30.00 | 2.7 | 2.7 | 3.5^5^, 3.5^6^ |
| V_2_SnC | V–C | 4 | 2.0526 | 1.02 | 0.05432 | 27.30 | 2.9 | 2.9 |  |
| Zr_2_SnC | Zr–C | 4 | 2.3118 | 1.05 | 0.01302 | 36.18 | 1.9 | 1.9 | 3.5^5^, 3.9^6^ |
| Nb_2_SnC | Nb–C | 4 | 2.2014 | 0.99 | 0.00139 | 31.98 | 2.3 | 2.3 | 3.8^6^, 3.5^6^ |
| Lu_2_SnC | Sn–C | 4 | 4.3478 | 0.12 | 0.00348 | 41.82 | 0.2 | 0.2 |  |
| Hf_2_SnC | Hf–C | 4 | 2.3158 | 1.39 | 0.00541 | 35.73 | 2.6 | 2.6 | 3.8^5^, 4.5^6^ |

**Table S6**. Sound velocities in km/s, Debye temperature and melting point in K, minimum and room temperature lattice thermal conductivity in W/m-K of Sc2SnC and existing M_2_SnC^2,4^ MAX phases.

| Phases | *ρ* | *v_l_* | *v_t_* | *v_m_* | *θ*_D_ | *T*_m_ | $\kappa_{\min}$ | *κ*_ph_ |
| --- | --- | --- | --- | --- | --- | --- | --- | --- |
| Sc_2_SnC | 5.005 | 6.017 | 3.593 | 3.976 | 449 | 1196 | 0.79 | 19.16 |
| Ti_2_SnC | 6.346 | 6.493 | 3.923 | 4.337 | 526 | 1550 | 0.99 | 30.10 |
| V_2_SnC | 7.073 | 6.013 | 3.420 | 3.801 | 473 | 1488 | 0.92 | 14.52 |
| Zr_2_SnC | 7.313 | 5.739 | 3.374 | 3.739 | 427 | 1384 | 0.76 | 20.68 |
| Nb_2_SnC | 8.369 | 5.761 | 3.182 | 3.546 | 422 | 1541 | 0.78 | 13.26 |
| Lu_2_SnC | 9.847 | 4.153 | 2.526 | 2.790 | 305 | 1152 | 0.52 | 15.61 |
| Hf_2_SnC | 11.796 | 4.691 | 2.702 | 3.000 | 346 | 1456 | 0.62 | 15.68 |

**Figure S1.** (a) Preferable vacancy sites for Sc, Sn and C; (b) preferable interstitial sites for Sc, Sn and C.

**References**

1. Li Youbing, Q. Y., Chen Ke, Chen Lu, Zhang Xiao, Ding Haoming, Li Mian, Zhang Yiming, Du Shiyu, Chai Zhifang, Huang Qing. Molten Salt Synthesis of Nanolaminated Sc_2_SnC MAX Phase. *Journal of Inorganic Materials* **36**, 773–778 (2021).
2. Hadi, M. A. *et al.* Chemically stable new MAX phase V_2_SnC: a damage and radiation tolerant TBC material. *RSC Adv.* **10**, 43783–43798 (2020).
3. Xu, Q. *et al.* Theoretical prediction, synthesis, and crystal structure determination of new MAX phase compound V_2_SnC. *Journal of Advanced Ceramics* **9**, 481–492 (2020).
4. Hadi, M. A., Kelaidis, N., Naqib, S. H., Chroneos, A. & Islam, A. K. M. A. Mechanical behaviors, lattice thermal conductivity and vibrational properties of a new MAX phase Lu_2_SnC. *Journal of Physics and Chemistry of Solids* **129**, 162–171 (2019).
5. Barsoum, M. W., Yaroschuk, G. & Tyagi, S. Fabrication and characterization of M_2_SnC (M = Ti, Zr, Hf and Nb). *Scripta Materialia* **37**, 1583–1591 (1997).
6. El-Raghy, T., Chakraborty, S. & Barsoum, M. W. Synthesis and characterization of Hf_2_PbC, Zr2PbC and M_2_SnC (M=Ti, Hf, Nb or Zr). *Journal of the European Ceramic Society* **20**, 2619–2625 (2000).
